# Iron imaging reveals tumor and metastasis macrophage hemosiderin deposits in breast cancer

**DOI:** 10.1371/journal.pone.0184765

**Published:** 2017-09-12

**Authors:** Avigdor Leftin, Nir Ben-Chetrit, Florian Klemm, Johanna A. Joyce, Jason A. Koutcher

**Affiliations:** 1 Department of Medical Physics Memorial Sloan Kettering Cancer Center, New York, New York, United States of America; 2 Cancer Biology and Genetics Program, Memorial Sloan Kettering Cancer Center, New York, New York, United States of America; 3 Department of Oncology, University of Lausanne, Lausanne, Switzerland; 4 Ludwig Institute of Cancer Research, University of Lausanne, Lausanne, Switzerland; 5 Department of Medicine, Memorial Sloan Kettering Cancer Center, New York, New York, United States of America; 6 Molecular Pharmacology Program, Memorial Sloan Kettering Cancer Center, New York, New York, United States of America; University of South Alabama Mitchell Cancer Institute, UNITED STATES

## Abstract

Iron-deposition is a metabolic biomarker of macrophages in both normal and pathological situations, but the presence of iron in tumor and metastasis-associated macrophages is not known. Here we mapped and quantified hemosiderin-laden macrophage (HLM) deposits in murine models of metastatic breast cancer using iron and macrophage histology, and in vivo MRI. Iron MRI detected high-iron pixel clusters in mammary tumors, lung metastasis, and brain metastasis as well as liver and spleen tissue known to contain the HLMs. Iron histology showed these regions to contain clustered macrophages identified by their common iron status and tissue-intrinsic association with other phenotypic macrophage markers. The in vivo MRI and ex vivo histological images were further processed to determine the frequencies and sizes of the iron deposits, and measure the number of HLMs in each deposit to estimate the in vivo MRI sensitivity for these cells. Hemosiderin accumulation is a macrophage biomarker and intrinsic contrast source for cellular MRI associated with the innate function of macrophages in iron metabolism systemically, and in metastatic cancer.

## Introduction

Macrophage accumulation is an emerging hallmark of the breast tumor and metastasis microenvironment [1–4]. At early stages of neoplasia, macrophages mount inflammatory response to initial tumor proliferation, and at later stages these macrophages adopt pro-tumor phenotypes associated with angiogenesis, metastatic dissemination, and immune-suppression [5, 6]. Histological evaluation of cells stained for macrophage surface receptor markers such as CD68 can provide a score of immune cell accumulation, or infiltration, that has been correlated with disease stage [7–12], and can also provide predictive information on expected outcomes of therapeutic intervention [13]. These assessments of tumor-associated macrophages (TAMs) are largely limited to biopsy of tumor tissue in situ, or sampling of lesions following bulk surgical resection. Collection of these specimens is routine, but invasive in primary breast tumors, and it becomes increasingly difficult to assess metastasis-associated macrophages (MAMs) in metastatic sites such as the lung and brain. Therefore, alternative non-invasive translational techniques are required that enable imaging and characterization of TAMs in vivo.

Macrophages function to recycle iron from senescent erythrocytes and have evolved to sequester the salvaged iron collected while mounting their innate immune response to tissue insult and pathogens [14–16]. To package their iron cargo, macrophages accumulate it in crystalline ferritin granule aggregates called hemosiderin.

So-called hemosiderin-laden macrophages (HLMs) also confer high MRI contrast and have long been used to detect macrophages preclinically and clinically [17–20]. Additionally, clinical MRI protocols have been developed that are sensitive to these cellular iron deposits [21, 22], and are similar to those utilized currently as standard-of-care breast tumor detection methods [23] suggesting that simultaneous tumor and HLM imaging can be performed.

As iron metabolism is a major area of research in breast and other cancers [24–28], but the association of hemosiderin deposits in tumor and metastasis has been relatively unexplored, we sought to determine the extent to which iron deposition occurs in the breast tumor and metastasis microenvironment, and evaluate our ability to map these deposits histologically and in vivo by MRI. We found that hemosiderin iron is indeed a metabolic biomarker of a population of macrophage deposits, and that the association of these iron(III)^+^ CD68^+^ macrophages with pro-inflammatory (allograft inflammatory factor-1,AIF1) and anti-inflammatory (mannose receptor, CD206) subsets varies according to tissue type. The association between hemosiderin and macrophages also provided a way to detect deposits of these cells in vivo by iron MRI with cellular level sensitivity. Using image feature detection algorithms we measured the frequency and size of the deposits in the iron MRI and histology. These findings show that iron deposition defines a metabolically distinct subset of immune infiltrates in tumors and metastasis, and provides an in vivo means to map and quantify the size of the deposits according to their innate iron status.

## Materials and methods

### Animal procedures

All animal work was approved by the Animal Care and Use Committee of MSKCC and performed according to their guidelines.

### Use of humane endpoints

The specific criteria used to determine when animals should be euthanized follow the humane endpoints for the cancer models used including: (a) primary tumor reached 1cm^3^, (b) first sign of respiratory distress due to lung metastasis, (c) first sign of neurological impairment, such as head tilting, changes in ambulation or disequilibrium due to brain metastasis. Humane endpoints were used in all studies, and once animals reached the humane endpoint they were euthanized immediately.

### Study design

A total of 8 mice bearing primary mammary tumors, 17 mice harboring lung metastases, and 19 mice displaying brain metastases were imaged upon presenting with signs associated with their humane endpoint to (1) confirm tumor burden as part of parallel investigations, and (2) to simultaneously map cellular iron deposits for the current studies. Tissues for ex vivo histology were available from 8 of the primary tumor models, 8 of the lung metastasis models, and 5 of the brain metastasis models due to sampling requirements for downstream analysis in parallel investigations. None of the animals died prior to, or after the current studies, and all animals were euthanized immediately after meeting humane endpoint criteria. Mice were inspected daily to minimize suffering and distress by veterinary staff and principal scientific investigators trained to identify clinical, physiological, and behavioral signs anticipated in the cancer models.

### Tumor models

In the primary tumor models, female 6 week-old FVB/n mice underwent orthotopic injection of 1 × 10^6^ syngeneic TS1 MMTV-PyMT tumor cells [29] suspended in 100μL of 50% Matrigel (BD Bioscience) into the lower mammary fat pad. MRI and histological measurements are made in tumors approximately 1cm^3^ in volume. In the lung metastasis models, female 6 week-old FVB/n mice underwent intravenous injection of 2.5 × 10^4^ TS1 MMTV PyMT tumor cells [29], and underwent MRI and histological analysis once presenting visible changes in respiration. In the brain metastasis models, female 6 week-old C57BL6 mice underwent intracardiac injection of 1×10^4^ brain metastasis cells obtained by propagation of the primary MMTV-PyMT mammary tumor line in 3D Matrigel culture followed by in vivo passage as brain metastasis [30], and were imaged by MRI and histology at first sign of neurological impairment.

### Bio-iron collection

Blood was withdrawn by vein puncture and collected in heparinized tubes. Crude hemosiderin was obtained from fresh mouse spleen by magnetic separation using MACS columns and running buffer (Miltenyi).

### Iron MRI

All MRI images were acquired with a 7T/30 cm horizontal bore MRI system (Biospec, Bruker Corp., MA) with a custom-built 30 mm inner-diameter transmit-receive quadrature radio-frequency coil. 2D multi-gradient echo (MGE) T_2_* relaxometry was used to acquire 16 multi-echo images with an inter-echo image acquisition time of 3.0ms, and an inter-scan time of 3s. Axial images were acquired with a slice thickness of 500μm and an in-plane pixel resolution of 0.01 mm^2^ (0.1 mm × 0.1 mm). Pixel-by-pixel determination of T_2_* relaxation times was performed by fitting of the magnitude of each pixel in the MGE image echo series with a standard mono-exponential fit function in Matlab (Natick, MA) and Fiji[31] [32]. T_2_* relaxation times were measured over the working range of iron(III) concentrations at 7T (0.0–0.3 mg iron(III) g^-1^ from iron(III) nitrate nonahydrate, Fisher Scientific). The linear relation between the relaxation rate R_2_* = 1/T_2_* and iron(III) concentration was found, and was subsequently used to generate the parametric iron(III) MRI maps. The iron(III) MRI maps were then stratified by concentration (total range, 0.0–0.3 mg g^-1^_;_ high, 0.15–0.3 mg g^-1^). Spatial characteristics of the high-iron(III) pixels were characterized by performing cluster analysis over the stratified iron MRI images using the Fiji Analyze Cluster tool. During the MRI scans mice were anesthetized with 1–3% isoflurane in O_2_ gas, and image acquisition was gated with the monitored respiration of the animal.

### Histology

Sections of PBS-perfused tissue were collected and fixed in 4% PFA for 24 hours at 4°C, and then washed with H_2_O, and re-suspended in 70% ethanol (Fisher Scientific). Tissues were paraffin embedded and 5μm sections cut onto glass slides for histology. The Prussian blue histochemical iron(III) assay was performed by placing de-parrafinized slides in a working solution of equal parts 5% potassium ferricyanide (Fisher Scientific) and 5% hydrochloric acid (Fisher Scientific) prepared in distilled water and stained for 30 minutes. Slides were then rinsed in distilled water, counter-stained with nuclear-fast red, and cover-slipped with Permount (Fisher Scientific). The immunohistochemical detection of CD68, and immunofluorescent detection of CD3, CD31, and CD68, AIF1, and CD206 was performed with a Discovery XT processor (Ventana Medical Systems). The tissue sections were de-parrafinized with EZPrep buffer (Ventana Medical Systems), antigen retrieval was performed with CC1 buffer (Ventana Medical Systems), and sections were blocked for 30 minutes with Background Buster solution (Innovex), followed by Avidin/biotin blocking for 8 minutes. For immunohistochemistry, anti-CD68 (Boster, cat# PA1518, 5ug/ml) was applied and sections were incubated for 5 hours, followed by 60 minutes incubation with biotinylated goat anti-rabbit antibodies (Vector Labs, cat#PK6101) at 1:200 dilution. The detection was performed with DAB detection kit (Ventana Medical Systems) according to manufacturer’s instructions. Slides were counterstained with hematoxylin and cover-slipped with Permount (Fisher Scientific). For immunofluorescence, anti-CD3 antibody (DAKO, cat# A0452, 1.2ug/ml), anti-CD31 (Dianova, cat# DIA-310 1ug/ml), anti-CD68 (Boster, cat# PA1518, 5ug/ml), anti-CD206 (Abcam, cat#ab64693, 1ug/ml), anti-AIF1 (Wako, cat#019–19741, 0.5ug/ml) were applied and sections were incubated for 5 hours, followed by 60 minutes incubation with biotinylated goat anti-rabbit antibodies (Vector Labs, cat#PK6101) for CD31, CD3, CD68, AIF1 and CD206 at 1:200 dilution. The detection was performed with Streptavidin-HRP D (part of DABMap kit, Ventana Medical Systems), followed by incubation with Tyramide Alexa Fluor A488 (Invitrogen, cat# T20922), TSA-Alexa 546 (Invitrogen, cat# T20933), TSA-Alexa 568 (Invitrogen, cat#T20914) TSA-Alexa 594 (Invitrogen, cat# T20935), or TSA-Alexa 647 (Invitrogen, cat# T20936) prepared according to manufacturer’s instructions with predetermined dilutions. After staining, slides were counterstained with DAPI (Sigma Aldrich, cat# D9542, 5 ug/ml) for 10 min, and coverslipped with Mowiol.

Slides were digitized with a Mirax Scan system and read with Panoramic Viewer (3DHISTECH, Budapest Hungary). Images were exported to Fiji, and pixels associated with macrophages were identified by their Prussian blue iron(III), DAB positive staining, or immunofluorescent positive labeling, and their size (>20μm^2^), and threshold masks of these pixels were generated using the Fiji Analyze Cluster tool. MRI resolution-matched iron(III) cluster maps were generated by resizing the threshold masks (1:100) by pixel averaging and bilinear interpolation with Fiji. The spatial characteristics of resolution-matched clusters were measured using the Fiji Analyze Cluster tool. The number of iron(III)^+^ macrophages per cluster was determined by using the cluster maps to define regions for cell counts in the full-resolution histological images. Metastasis surveys were conducted in a similar manner, with the exception that the lesions were first identified automatically by thresholding the neutral red counter-stained Prussian Blue tissue sections to identify individual lesions, and within these lesions, we performed the macrophage counts and resolution-matched cluster quantification using the Fiji Analyze Cluster tool.

### Statistics

Results are presented as mean ± s.e.m. unless otherwise indicated. Comparison between groups and distributions was performed using two-tailed unpaired student’s t-tests, or Mann-Whitney tests as appropriate. All statistical calculations were performed in GraphPad Prism 7 Software.

## Results

**Fig 1:**
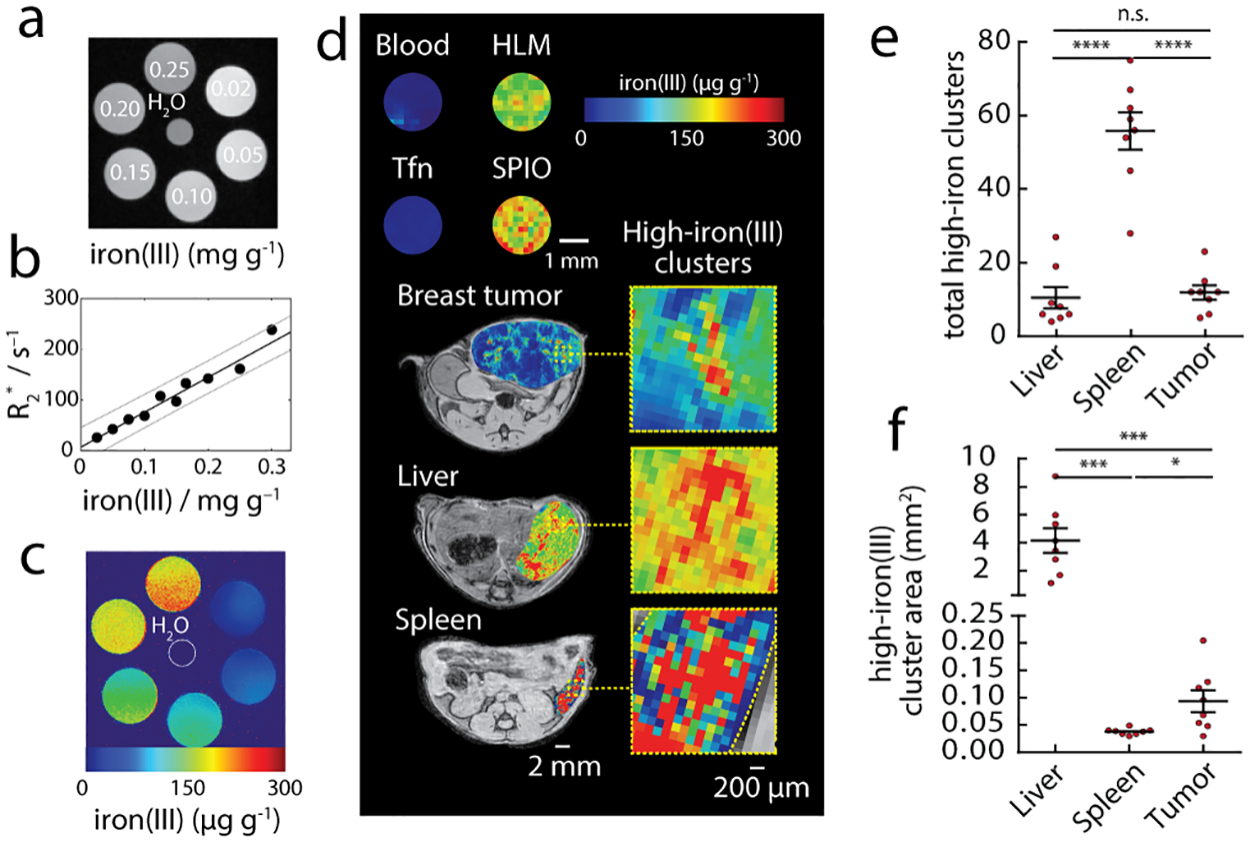
Quantitative contrast agent-free MRI of macrophage iron deposits. (a) Multi-gradient echo (MGE) MRI image (first-echo) showing variation of contrast with increasing concentration of iron(III) (0-0-0.25 mg g^-1^). (b) Quantitative MRI measurements of signal relaxation rates R_2_* (= 1/ T_2_*) for the MGE image series as a function of iron(III) concentration (solid-line linear fit, dashed lines 95% confidence interval). (c) Parametric iron(III) maps generated for the standards, and H_2_O (dashed circle). (d) Iron MRI maps of whole blood, transferrin (Tfn, ~20μg iron/mL), macrophage hemosiderin from mouse spleen, superparamagnetic iron oxide (SPIO) nanoparticles in aqueous solution (200μg/mL), and in vivo in mammary tumors, livers (no tumor), and spleens (no tumor) of the MMTV-PyMT breast cancer models. Upper scale bar, 1mm, lower scale bar 2mm. Expansions show high-iron pixel cluster regions indicative of hemosiderin deposition. Scale bar 200μm. High-iron(III) MRI clusters quantified automatically by (e) counting the total number of high iron pixel clusters in the iron MRI images (0.15–0.3 mg iron(III) g^-1^ range), and (f) measuring the average cross-sectional areas of the clusters (mm^2^). Points are individual animals (mean±s.e.m.; n = 8 animals; n.s. not significant p>0.05, *p<0.05, **p<0.01, ***p<0.001, ****p<0.0001 two-tailed unpaired t-test).

Multi-gradient echo (MGE) T_2_*-weighted MRI exhibits reduced pixel intensity with increasing concentration of iron(III) (Fig 1a). As a quantitative measure of this sensitivity to iron, we calculated the rate of MGE image pixel decay (R_2_* = 1/T_2_*) in solutions of increasing iron concentrations and found a positive linear relation between these experimental variables (Fig 1b). Pixel-wise parametric maps of iron concentration were generated using this relation for the iron standards over the full range of the iron(III) contrast at 7T (Fig 1c, 0–0.3 mg iron g^-1^). When applied to other bio-iron sources this iron mapping method confirmed that iron levels of blood, and iron(III)-transferrin (~20μg transferrin iron g^-1^) were at the lower limit of the detectable range of the iron MRI maps (Fig 1c). [33–35]. By contrast, superparamagnetic hemosiderin from spleen [36] [37, 38] and iron nanoparticles mimicking the bio-iron deposits (200μg iron g^-1^) were found in the high-iron range.

These tests demonstrated the ability to map iron in vivo, and further suggested the ability to differentiate between different bio-iron sources using in vivo MRI. We then imaged orthotopic MMTV-PyMT mammary tumors, livers, and spleens in vivo (Fig 1d). The iron MRI examinations revealed “hot-spot” high-iron(III) pixel clusters that were quantified by automated image cluster analysis of high-iron stratified MRI maps (0.15–0.3 mg iron(III) g^-1^) to measure the size (Fig 1e) and frequency of each of the clusters in the various tissues (Fig 1f). The total number and size of the high-iron(III) clusters varied significantly between tissue types suggesting different quantities, and different spatial distributions of HLMs. High-iron clusters were found sparsely in discrete deposits around peritumoral regions. In the liver, large clusters suggested the known distribution iron-laden Kuppfer cells throughout the liver. In the spleen where HLMs are densely deposited in the red-pulp, focal high-iron(III) clusters were significantly more frequent than both tumors (p<0.0001) and liver (p<0.0001).

**Fig 2:**
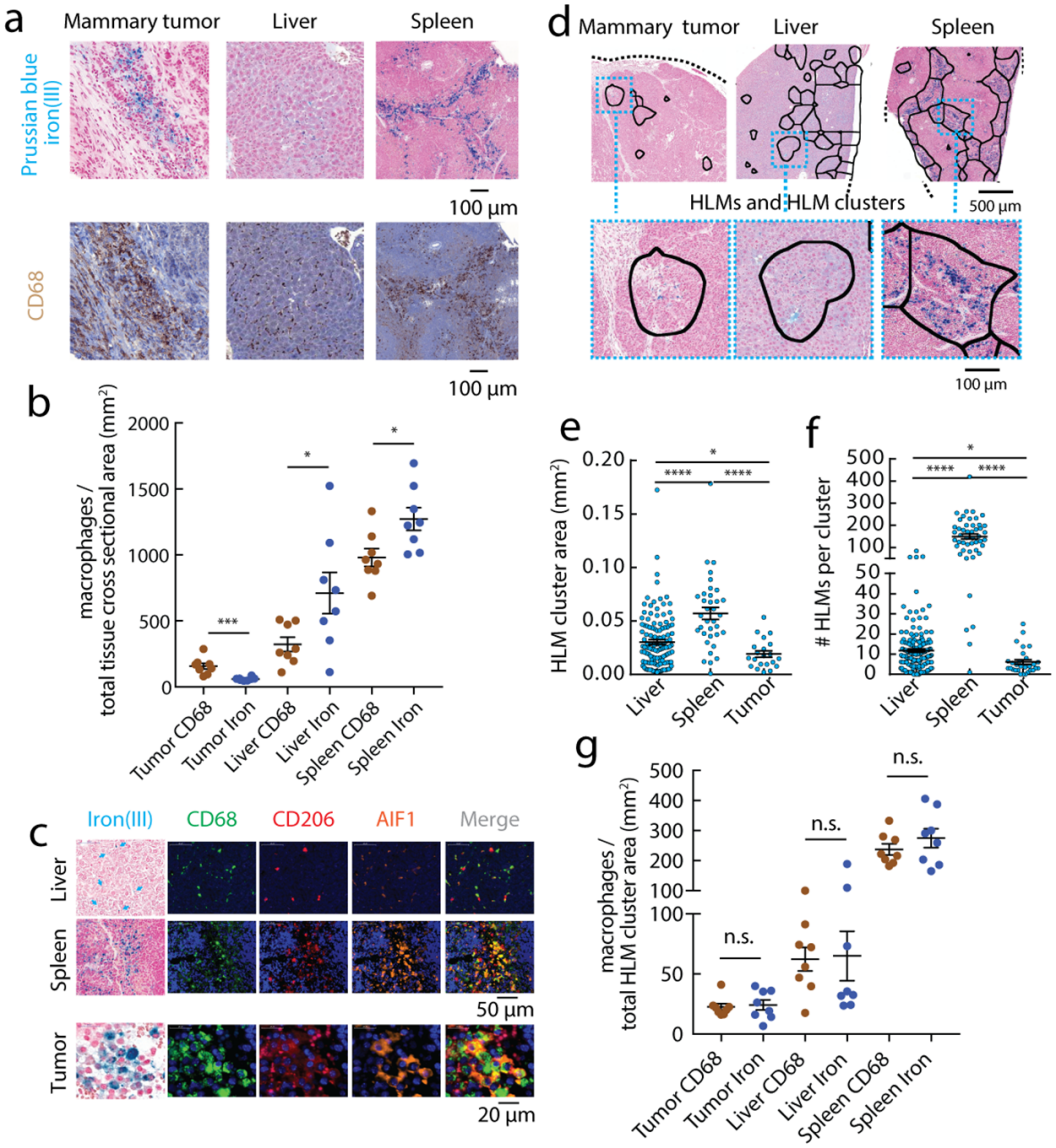
Hemosiderin iron is a biomarker of macrophage deposits in systemic and tumor microenvironments. (a) Prussian blue iron(III) and CD68 macrophage histology from orthotopic MMTV-PyMT mammary tumor, liver, and spleen. Scale bar 200μm. (b) The total number of Prussian blue iron(III)^+^ and CD68^+^ macrophages per mm^2^ of the whole tissue cross-sectional area measured by automated cell counting. Points are individual animals (mean±s.e.m.; n = 8 animals; n.s., not significant *p<0.05, ***p<0.001 two-tailed unpaired t-test). (c) Iron(III) histochemistry beside single color channel and merged CD68, CD206, and AIF1 immunofluorescent markers. Liver (arrows denote iron(III)^+^ features) and spleen scale bar 50μm, mammary tumor scale bar 20μm. (d) The Prussian blue iron(III) images of MMTV-PyMT mammary tumors, livers, and spleens processed by down-sampling the resolution of the digitized histology to the MRI pixel scale (1:100, 0.01mm^2^/pixel) to generate resolution-matched clusters centered around iron(III)^+^ HLM deposits (solid lines). Scale bar 100μm. Automated size distribution analysis of (e) cross-sectional area of the clusters, and (f) frequency of the resolution-matched HLM clusters. Each point is a cluster detected in whole cross-sections of orthotopic MMTV-PyMT mammary tumors, livers, and spleens (mean±s.e.m.; n = 8 animals; *p<0.05, **p<0.01, ***p<0.001, ****p<0.0001 Mann-Whitney test). (g) The number of Prussian blue iron(III)^+^ and CD68^+^ macrophages per mm^2^ of the total cluster area. Points are individual animals (mean±s.e.m.; n = 8 animals; n.s., not significant *p<0.05 two-tailed unpaired t-test).

To further confirm the presence of these HLMs at cellular resolution, whole tissue cross-sections of orthotopic murine MMTV-PyMT mammary tumors, livers, and spleens were stained by Prussian blue iron(IIII) and the general marker CD68 (Fig 2a), revealing iron(III)^+^ cells characterized as HLMs co-localized with CD68^+^ macrophages. Counting the total number of iron(III)^+^ macrophages and CD68^+^ macrophages per mm^2^ of the whole tissue cross-section area (Fig 2b) showed both total HLMs and CD68 macrophages increased in frequency from tumor<liver<spleen. The relative numbers of iron(III)^+^ HLMs to CD68^+^ macrophages was slightly greater for Kuppfer cells (p<0.05) and red-pulp macrophages (p<0.05) of the reticuloendothelial organs; iron-laden populations were less frequent than CD68 infiltrates in the tumors (p<0.001). Additional immunofluorescence staining was performed to assess the association of the iron(III)^+^ cells with other macrophage subsets at cellular resolution, confirming the immunohistochemical determination of the co-localization with CD68, and further revealing that these macrophages also express both pro-inflammatory (AIF1^+^, allograft inflammatory factor 1) and anti-inflammatory (CD206^+^, mannose receptor) markers in the mammary tumors, livers, and spleens (Fig 2c). These measurements indicate that at the whole tumor level of analysis, macrophages determined by their association with iron comprised a unique population present systemically, and a unique sub-population of the macrophage subsets found in the tumors.

To estimate the contributions of the HLM deposits to the high-iron(III) MRI clusters, all HLMs in the Prussian Blue stained cross-sections were automatically detected, and thresholded images of these cellular deposits were downsampled to the MRI pixel dimensions (1:100 pixel resolution scale factor) to generate “resolution-matched” HLM clusters in the histology (Fig 2d). These regions were quantified by the same image cluster analysis as the iron MRI yielding the cluster areas (Fig 2e), and high-resolution counts of HLMs within these regions (Fig 2f). HLM clusters ranged from approximately 0.01–0.1 mm^2^ (on the order of 1–10 MRI pixel^2^), and the number of macrophages per deposit ranged from approximately 10 HLMs/cluster in the mammary tumors to >100 HLMs/cluster in the spleen. The total iron(III)^+^ and CD68^+^ macrophages in the deposits were found to be the same in all the tissues (P>0.05) (Fig 2g), and increased from breast<liver<spleen as in the whole cross-section histological scores. This proves the association of macrophages with hemosiderin iron in the deposit regions, and further corroborates the iron MRI measurements by confirming the location, size, and cellularity of the HLM deposits in the tissues.

**Fig 3:**
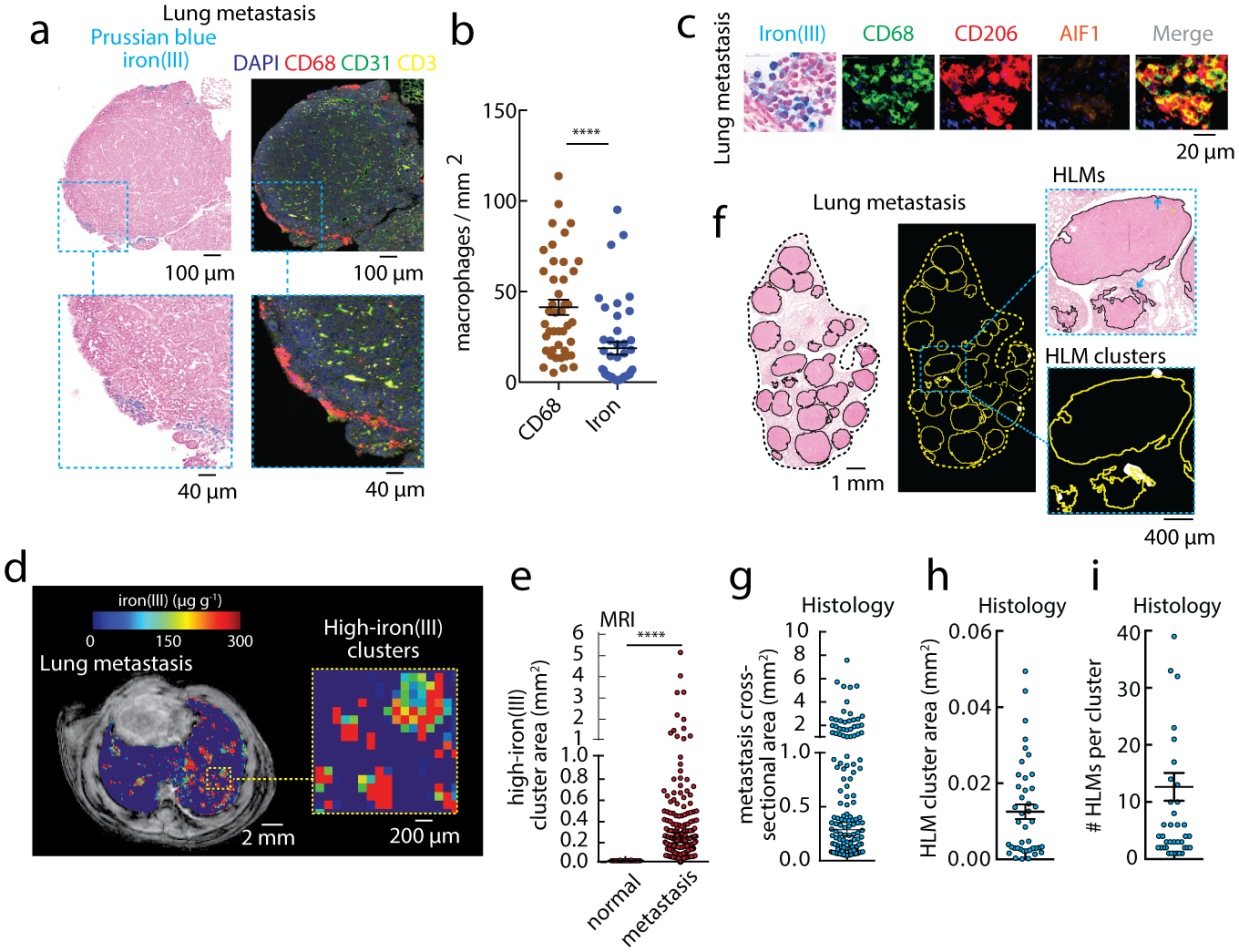
Imaging HLM deposits in lung metastasis. (a, left) Prussian blue iron(III) histology, and (right) immunofluorescence staining (DAPI, CD68, CD31, CD3) of lung metastasis from MMTV-PyMT mammary-lung model (intravenous injection). Scale bar 100μm. Expansion shows HLM deposits. Scale bar 40μm. (b) The total number of iron(III)^+^ HLMs and CD68^+^ macrophages per mm^2^ lung metastasis measured by automated cell counting. Points are individual metastases. (mean±s.e.m.; n = 42 metastases, n = 8 lung sections; ****p<0.0001 Mann-Whitney test). (c) Iron(III) histochemistry beside single color channel and merged CD68, CD206, and AIF1 immunofluorescent markers. Scale bar 20μm. (d) In vivo iron(III) MRI maps measured in MMTV-PyMT mammary-lung metastasis models. Scale bare 2mm. Expansion shows high-iron(III) cluster regions and individual metastases. Scale bar 200μm. (e) High-iron(III) MRI clusters automatically analyzed to determine the average cross-sectional area of the clusters (mm^2^). Points are individual high-iron(III) clusters measured in metastasis and normal lung regions (mean±s.e.m.; n = 166 metastases, n = 47 normal lung regions, n = 17 mice; ****p<0.0001 Mann-Whitney test). (f) Whole lung cross sections of the metastasis models stained for Prussian blue iron(III), and processed images showing individual metastasis (outlines), HLM deposits (arrows), and resolution-matched HLM clusters in the individual metastasis (filled regions). Scale bar 1mm. Expansion scale bar 400μm. Prussian blue iron(III) histology automatically assessed for (g) metastasis cross-sectional area (mean±s.e.m. n = 169 metastases, n = 8 lung sections), (h) MRI-resolution matched HLM cluster area, and (i) number of iron(III)^+^ HLMs per cluster (mean±s.e.m.; n = 42 iron(III)^+^ metastases out of 169 counted, n = 8 lung sections).

As HLMs were detected in primary tumors, we hypothesized that we could also detect them in advanced stages of cancer such as lung metastases. We initiated an intravenous injection model of MMTV-PyMT mammary-to-lung metastasis, and imaged the HLM deposits by histology and in vivo by iron MRI. Immunofluorescence detected CD68^+^ macrophages at the same lesion margins as iron(III)^+^ HLMs stained by Prussian blue. These cells were found in the proximity of CD31^+^ vasculature, where CD3^+^ T-cells were also localized indicating a functional tumor immune microenvironment rather than necrotic regions (Fig 3a). The number of iron(III)^+^ HLMs was significantly smaller than CD68^+^ macrophage counts per mm^2^ lung metastases showing that in this metastatic setting, they are a sub-population of the total infiltrating macrophages (p<0.0001) (Fig 3b). At cellular resolution, these HLMs were found to express high levels of CD68 and CD206 with either no or low expression of AIF1 indicating that they are primarily acting in an anti-inflammatory capacity in the lung metastases (Fig 3c). The histological analysis also revealed that the iron(III)^+^ macrophage deposits occurred in only 24% of the lesions detected (42 iron(III)^+^ metastases / 169 total metastases), indicating that HLMs are a subpopulation of macrophages in lung metastasis.

In vivo iron(III) MRI examinations were also performed in the lung metastasis models. Iron(III) maps revealed numerous high-iron(III) clusters (Fig 3d), and image segmentation and automatic cluster analysis of the high-iron(III) regions revealed a highly-significant association of clusters with metastases (p<0.0001, Fig 3e). However, statistical analysis of the high-iron cluster distribution revealed large areas that we could not rationalize to be discrete deposits of macrophages based on our findings in livers, spleens, and mammary tumors. This indicated to us the that the known difficulties using MRI to image air-tissue interfaces in the lung [39] instead led to high-iron clusters delocalized to the size to each metastasis rather than detecting small HLM deposits within the metastatic lesions. Measurement of lung metastasis areas from histology confirmed that the cluster sizes measured by iron(III) MRI in lung were indeed on the order of the metastatic lesions themselves (Fig 3f and 3g). Nonetheless, we performed the resolution-matched HLM cluster assessment to demonstrate the potential of the method if more tractable lung MRI conditions were available, and indeed the HLM cluster areas (Fig 3h), and the counts of HLMs in these discrete regions were on the order of those measured in primary tumors (Fig 3i). These measurements indicate that while hemosiderin deposition and clustering is a feature of CD68^+^ CD206^+^ macrophages in lung metastasis, it is not detectable in a straightforward application of the iron MRI measurements because of the constraint that magnetic susceptibility mismatch imposes on MRI measurements of the lung.

**Fig 4:**
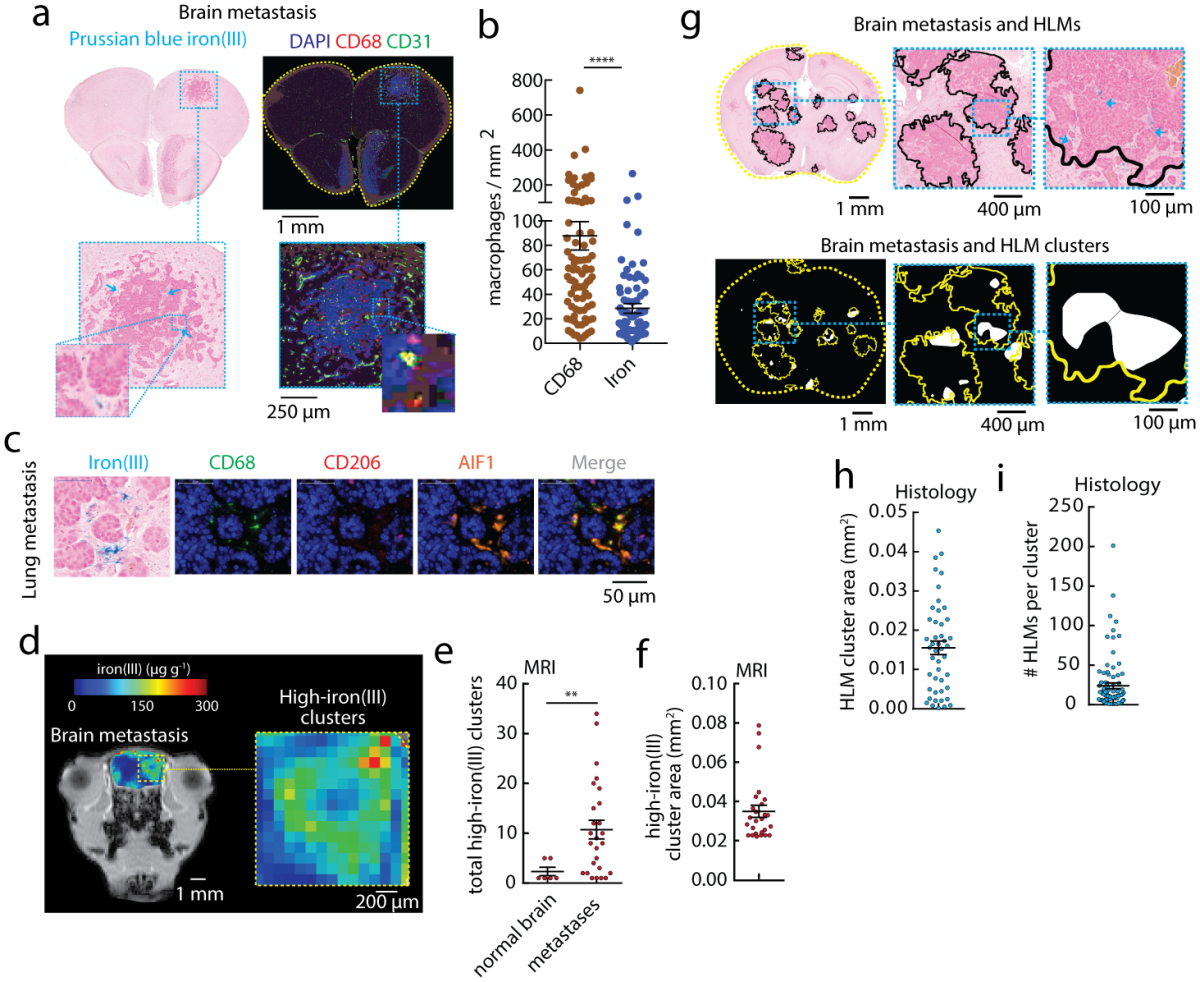
Imaging HLM deposits in brain metastasis. (a, left) Prussian blue iron(III) histology, and (right) immunofluorescence staining (DAPI, CD68, CD31) of brain metastasis from MMTV-PyMT mammary-brain model (intracardiac injection). Scale bar 1 mm. Expansion shows individual metastasis, and individual iron(III)^+^ HLM deposit (5x). Scale bar 250μm. (b) The total number of iron(III)^+^ HLMs and CD68^+^ macrophages per mm^2^ brain metastasis measured by automated cell counting. Points are individual metastases. (mean±s.e.m.; n = 86 iron(III)^+^ and CD68^+^ metastasis; n = 16 brain sections, n = 5 animals; ****p<0.0001 Mann-Whitney test). (c) Iron(III) histochemistry beside single color channel and merged CD68, CD206, and AIF1 immunofluorescent markers. Scale bar 50μm. (d) In vivo iron(III) MRI maps measured in MMTV-PyMT mammary-brain metastasis models. Scale bar 2mm. Expansion shows high-iron(III) cluster regions and individual metastases. (e) High-iron(III) MRI clusters automatically counted in normal brain regions and the metastases, and (f) the average cross-sectional areas of the high-iron(III) clusters (mean±s.e.m.; n = 97 metastasis, n = 26 high-iron(III) clusters; n = 66 normal regions, n = 6 high-iron(III) clusters, n = 19 animals; **p<0.001 Mann-Whitney test). (g) Whole brain cross sections of the metastasis models stained for Prussian blue iron(III). The processed images show individual metastasis (outlines), HLM deposits (arrows), and MRI resolution-matched HLM clusters in the individual metastasis (filled regions). Scale bar 1mm. Expansion scale bar 400μm. Prussian blue iron(III) histology automatically assessed for (h) the average HLM cluster cross-sectional area measured per brain metastasis, and (i) number of iron(III)^+^ HLMs per cluster (mean±s.e.m.; n = 46 brain metastasis; n = 212 HLM clusters, n = 16 brain sections, n = 5 animals).

To investigate HLM deposition in another metastatic setting, we used an in vivo MMTV-PyMT brain metastasis model. Tumor cells were introduced by intracardiac injection, and we mapped HLMs in the brain metastases by histology and iron MRI. Prussian blue histology confirmed iron(III)^+^ HLM deposits at the edges of the invasive metastatic lesions (Fig 4a), and immunofluorescence revealed these macrophages to be a sub-population of the CD68^+^ macrophages as in the other breast cancer tissues (p<0.0001) (Fig 4b). In the brain metastasis, these iron^+^ CD68^+^ cells also expressed high levels of inflammatory AIF1, and no or very low levels of CD206 (Fig 4c) suggesting that they are activated microglia. These iron(III)^+^ cells were also present in the majority ~81% of the lesions (86 iron(III)^+^ metastases /105 total metastases). Iron MRI examinations revealed focal small high-iron(III) MRI pixel clusters associated with HLM deposits, and elevations in low level contrast that distinguished brain metastases from tissue background (Fig 4d). Counts of high-iron(III) clusters in these metastases compared to normal brain regions revealed a significant association of these clusters with the lesions (p<0.01, Fig 4e). Areas of the high-iron clusters (Fig 4f) were consistent with the small cluster sizes of mammary tumors and spleens, and HLM clusters generated automatically within each metastasis from histology (Fig 4g) revealed that HLM cluster areas (Fig 4h) were also on the order of the in vivo measurements, and measuring the number of HLMs per cluster by these parallel histological assessments (Fig 4i) support the use of iron MRI in mapping HLM deposition in brain metastasis with cellular sensitivity.

## Discussion and conclusions

The association of iron deposits with macrophages underscores their metabolic role in recycling iron for heme biosynthesis. The function of macrophages in storing iron is highlighted in the livers and spleens, organs known to harbor HLMs and are central to iron homeostasis. Here, iron(III)^+^ and CD68^+^ macrophages occurred with similar frequency, and were also associated with AIF1 and CD206 populations indicating that most of the macrophages were engaged in iron-handling roles. In mammary tumors, however, iron(III)^+^ macrophages were relatively fewer than in the liver and spleen, and only a fraction of the HLMs were associated with the total CD68^+^, AIF1^+^, and CD206^+^ macrophages also found in the mammary tumors. Similarly, in lung and brain metastases, HLM populations were even more rare, presumably because of the restrictive cellular barriers (blood-brain/lung) that limit deposition of immune cells and erythrocytes, and thus limits accumulation of iron in macrophages in these organs [40–42]. These observations indicate that while the iron deposition in tumor and metastasis associated macrophages is not as prominent as in organs such as the spleen and liver, iron storage in hemosiderin is an important conserved function for macrophages in both those iron-handling tissues and in the tumor and metastatic microenvironments.

In the innate immune response, resident macrophages and circulating myeloid cells from bone marrow respond to and transmit cytokine cues signaling inflammatory and anti-inflammatory programs, leading to adaptive signaling and vascular remodeling of the tumor microenvironment [43–45]. In these situations macrophages can change their surface receptor expression giving rise to so-called polarization in a given tissue setting [5, 46]. The surface receptor CD68 is a pan-macrophage marker and is used commonly for both pre-clinical and clinical assessment of macrophage infiltration, and thus, the majority of our studies relied on this marker to evaluate the iron staining in relation to this general macrophage population. We also assessed the association of the HLMs with other specialized functional markers including AIF1 (pro-inflammatory, M1) and CD206 (anti-inflammatory, M2). We found that in mammary tumors, livers, and spleens, iron(III)^+^ CD68^+^ HLMs expressed both AIF1 and CD206 to a similar degree indicating that in these tissues the HLM subpopulations were not polarized to either phenotypic extreme. In metastasis, however, HLMs were strongly polarized. The iron^+^ CD68^+^ HLMs in lung metastasis exhibited high levels of CD206 not AIF1, and in brain iron^+^ CD68^+^ HLMs were predominantly associated with high levels of AIF1, not CD206. Interestingly, this demonstrates that while our imaging investigations identify specific populations of macrophages according to their hemosiderin deposits in all the tissues studied, the association of the iron-laden macrophage deposits with the phenotypic polarization markers appears to be related more to the tissue where they are found, rather than the association with iron deposition alone.

Mapping iron deposit size and frequency provides a new application of iron MRI techniques that enables the localization and quantification of macrophage deposits systemically, and in cancer microenvironments. As the basic iron MRI experiments are commonly performed clinically, and the new spatial image analysis approaches rely on simple cluster map processing procedures, their combination has high translational potential. Applications for the iron MRI and cluster mapping methods include the quantification of cellular distributions following iron-oxide contrast agent injections[47, 48], monitoring macrophage depletion using injections of clodronate liposomes[49], or tracking cellular response to administration of macrophage targeted cancer therapies [48, 50, 51].

The studies presented here link a hallmark phenotype of macrophages, hemosiderin iron deposition, with the presence of infiltrating macrophages in metastatic breast cancer models, and we describe an in vivo approach for the quantitative mapping of these HLM deposits and measurement of their frequency and size. Taken together, imaging macrophages according to their endogenous iron levels reveals a cellular biomarker associated with their innate role in iron metabolism, and bridges a gap in our ability to quantitatively assess macrophage accumulation in vivo.
